# Successful endoscopic treatment of Mirizzi syndrome type V complicated with a cholecystocolic fistula

**DOI:** 10.1055/a-2619-7019

**Published:** 2025-07-01

**Authors:** Kazunari Nakahara, Ema Takenouchi, Yusuke Satta, Yu Matsuda, Yosuke Igarashi, Junya Sato, Keisuke Tateishi

**Affiliations:** 1 Department of Gastroenterology, St. Marianna University School of Medicine, Kawasaki, Japan

Mirizzi syndrome complicated with a cholecystocolic fistula (CCF) is classified as Mirizzi syndrome type V 1 . Although surgical management with cholecystectomy, fistula takedown, and possible colonic resection is indicated as the standard treatment for Mirizzi syndrome complicated with CCF 2 3 4 , we describe successful management of such a case using endoscopic therapy alone ( Video 1 ).

Successful complete endoscopic stone removal and cholecystocolic fistula closure for Mirizzi syndrome type V.Video 1

A 50-year-old man was admitted to our hospital with jaundice. Magnetic resonance cholangiopancreatography revealed stones filling the gallbladder and causing a stricture of the hilar bile duct, resulting in the diagnosis of Mirizzi syndrome ( Fig. 1 ). We performed endoscopic retrograde cholangiopancreatography (ERCP). Cholangiography revealed the hilar biliary stricture due to gallbladder stones and contrast medium flowed from the hilar bile duct into the gallbladder, resulting in the diagnosis of a cholecystobiliary fistula ( Fig. 2 ). A nasobiliary drain was placed in the intrahepatic bile duct, and after the improvement of jaundice, ERCP was repeated. Peroral cholangioscopy revealed gallbladder stones protruding into the hilar bile duct through the cholecystobiliary fistula ( Fig. 3 ), and electrohydraulic lithotripsy (EHL) was performed. Five ERCP with EHL sessions resulted in the complete removal of the gallbladder stones. However, cholecystography revealed that the CCF and colon were contrasted ( Fig. 4 **a** ). Cholangioscopy-guided biopsies of the CCF revealed no malignancy. Therefore, we inserted a guidewire through the CCF into the colon ( Fig. 4 **b** ) and placed a nasal catheter in the colon ( Fig. 4 **c** ). We then performed a colonoscopy through which the CCF was easily detected at the hepatic flexure using the placed nasal catheter as a marker ( Fig. 5 **a** ). After removing the nasal catheter, the CCF was successfully closed with a large grasping clip (MANTIS Clip; Boston Scientific, Marlborough, Massachusetts, USA) ( Fig. 5 **b** ). Complete endoscopic removal of gallbladder stones and endoscopic CCF closure were successfully performed for Mirizzi syndrome type V complicated with CCF, thus avoiding surgery.

**Fig. 1:**
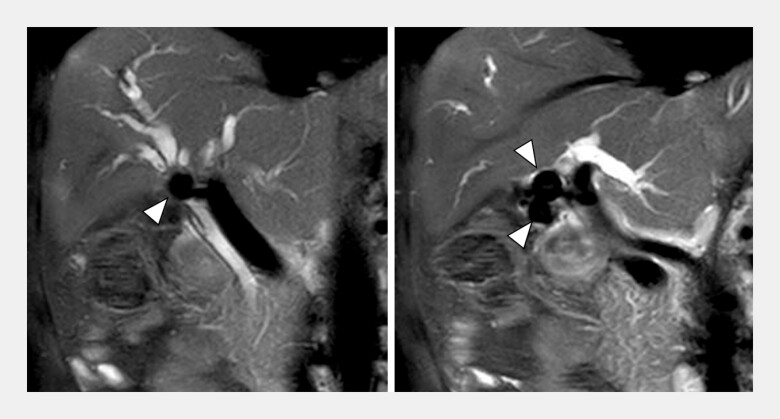
Magnetic resonance cholangiopancreatography revealed stones filling the gallbladder and causing a stricture of the hilar bile duct.

**Fig. 2:**
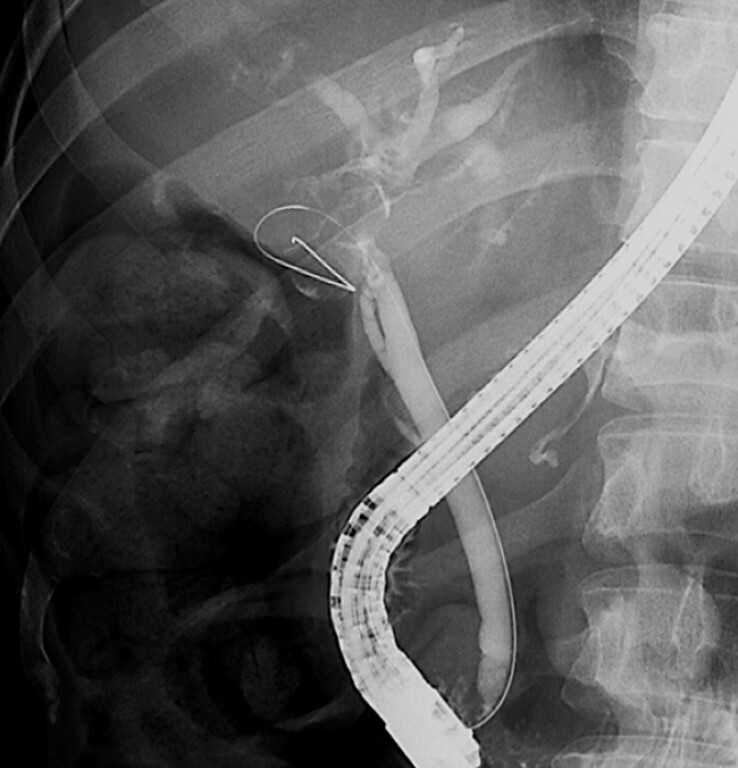
Cholangiography revealed the hilar biliary stricture due to gallbladder stones and inflow of contrast medium from the hilar bile duct into the gallbladder. A guidewire was inserted from the hilar bile duct into the gallbladder, indicating the cholecystobiliary fistula.

**Fig. 3:**
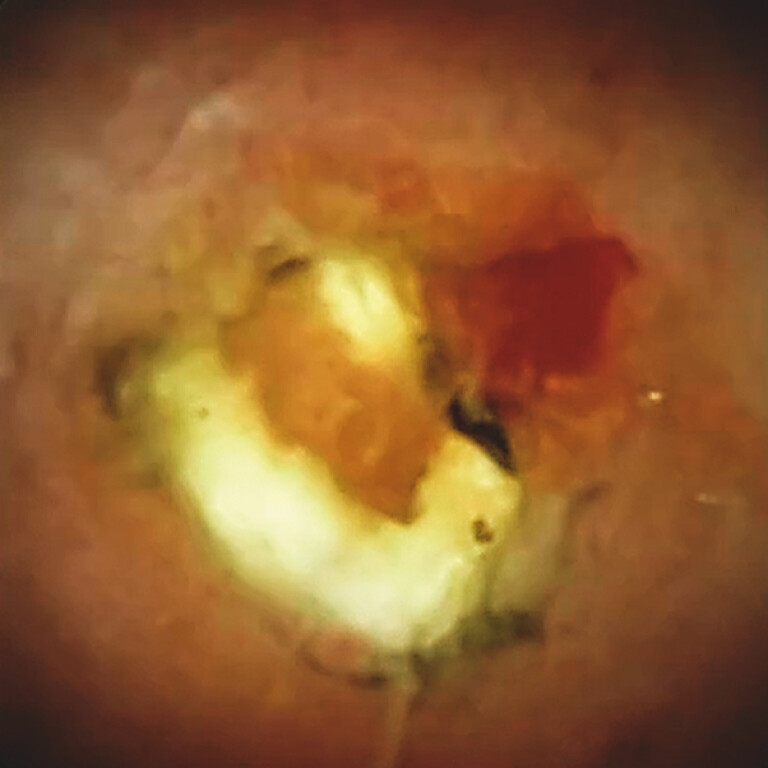
Cholangioscopy revealed a stone protruding from the gallbladder through the cholecystobiliary fistula into the hilar bile duct.

**Fig. 4:**
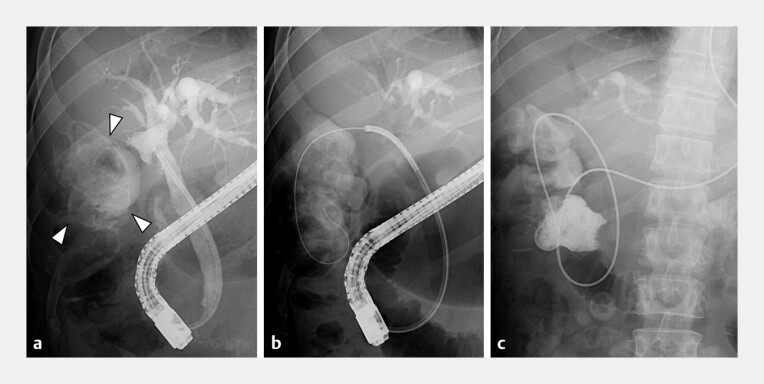
**a** Cholecystography revealed the cholecystocolic fistula, and the colon was contrasted (arrowheads). **b** A guidewire was inserted through the cholecystocolic fistula into the colon. **c** A nasal catheter was placed in the colon.

**Fig. 5:**
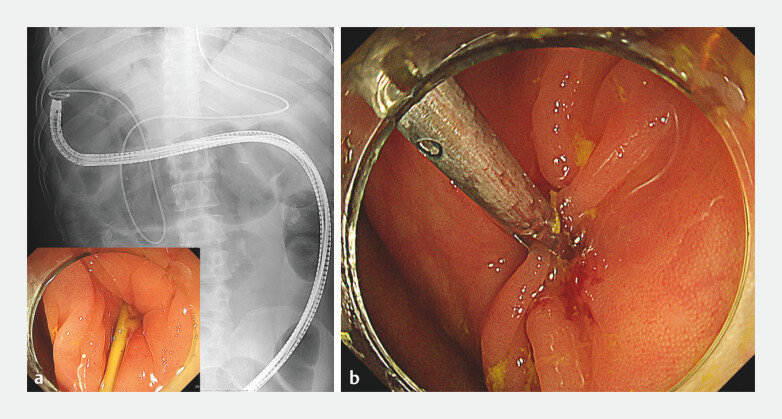
**a** The cholecystocolic fistula at the hepatic flexure was easily identified during colonoscopy with the nasal catheter placed as a marker. **b** After removing the nasal catheter, the cholecystocolic fistula was successfully closed with a large grasping clip.

Endoscopy_UCTN_Code_TTT_1AR_2AH

## Notes

Endoscopy E-Videos https://eref.thieme.de/e-videos
*E-Videos* is an open access online section of the journal *Endoscopy* , reporting on interesting cases and new techniques in gastroenterological endoscopy. All papers include a high-quality video and are published with a Creative Commons CC-BY license. Endoscopy E-Videos qualify for HINARI discounts and waivers and eligibility is automatically checked during the submission process. We grant 100% waivers to articles whose corresponding authors are based in Group A countries and 50% waivers to those who are based in Group B countries as classified by Research4Life (see: https://www.research4life.org/access/eligibility/ ). This section has its own submission website at https://mc.manuscriptcentral.com/e-videos .
